# The M. V. A., and Yours Respectfully

**Published:** 1876-07

**Authors:** Geo. Watt


### DENTAL REGISTER.

Vol. XXX.] JULY, 1876. [No. 7.

### THE M. V. A., AND YOURS RESPECTFULLY.

BY DR. GEO. WATT.

*Read before the Mississippi Valley Dental Society, upon retiring from the presidency, March 2d, 1876.*

That historical fly, which from the axle of the stage coach, boasted of the big dust kicked up by himself and the horses, was, after all, not much of a philosopher. Progress, not dust, was the aim; then why not boast of speed, for had he not undoubtedly kept up? Had he made this judicious boast, he would, at once, rank as a leading character, for, as the car of progress makes its steady onward journey, how many ingloriously fall behind! All honor then to the ambitious, progressive fly! If called for, he was at hand. His more slothful comrades dissuaded him. What can a fly do on a journey, or at its end, said they. I'll go and see said he. And he went. Would it not be well for members of our profession, of our societies, and especially of this association, to do likewise? Can't add anything of interest, you say? The fly was of but little force to the team, yet he was willing to go. But if you can't add perhaps you can subtract. The fly could. With four warm horses in front, he was content, on the axle, in reference to his commissary department.

Our good old coach, the "M. V. A.," had been well started on her journey, and was just reaching her eighth mile post when "Yours Respectfully" first caught sight of her. She was drawn by Goddard, McCullum, Bonsall, Baxter, A. M., Hunt, Ulrey, James Taylor, Van Emon, A. M. Leslie, J. Taft. Her first consciousness of the existence of the insect is recorded as follows: "Drs. Watt and Hamill were present by invitation." HAMILL! the genial, volatile, hopeful, solid and manly specimen of professional energy! How strange that he has been so long at rest, while his that day comrade remains to waste your time in dry reminiscence!

But, to drop all figures, how much to gratify, how much cause for thankfulness have we, in the fact that so many members present that day, have been ever since, and are now, active, earnest workers in and through this mother of societies, for the upbuilding of our profession, and for the good of humanity. And, with a tear for the departed, let us strive to imitate their bright examples, engraven on our memories, and recorded in the proceedings of the society they loved. So well do I remember our brothers, A. M. Hunt and A. M. Leslie as the most active workers at that meeting.

At this meeting an affair occurred of some historical interest, an account of which, by unanimous vote, was totally excluded from the minutes. It may be seen that, on calling the roll, the first day, at 10 a. m., but five members were present. The absentees included the president, the 1st and 2d vice-presidents, the recording secretary, the executive committee, etc. This was certainly very disheartening. Accordingly, a special committee was appointed to report next day on the state of affairs. If my memory is correct Dr. Goddard was chairman of this committee. The report recommended adjournment *sine die*. This elicited a warm discussion, Dr. Leslie leading the opposition to the adoption of the report. After a very full consideration, the committee asked leave to withdraw the report which was granted. It was during this suicidal struggle that my friend Hamill and I were received as members; and, in view of the efficiency of the Association in advancing the welfare of our profession, and humanity in general, my mind often recurs to this scene. Had that report been adopted and this old society disbanded, who can picture the consequences? This, the oldest society in our profession, has guided the formation of others, all which have been at all efficient in advancing the science of our profession, and elevating its membership, having mainly copied its leading characteristics. The good boy's ideal of a true woman, is his own good mother; and the beautiful, blushing maiden who possesses the greatest number of traits of her character, draws his heart's best affections to herself as sure as the trellis attracts the tendrils of the vine. So the many sons of our good old mother, so well known as Missis. V. A., have attracted only such societies as already possessed, or were readily molded into her image. It is probable her range of usefulness has been wider in this direction than in any other. Who of her active members needs time to familiarize himself with the workings of any other leading society? With the routine business transacted, they, with one consent, fall into the line of essays, oral discussions, examination of instruments, specimens, etc., following the example of the mother of societies, as closely as the well trained daughter copies her mother's housekeeping.

No man of ordinary mental susceptibility could be an active member, or even an attentive member, of such a society as this, without growth of mental and moral energy. How vividly this is impressed upon the mind of the writer, words can not adequately describe. If he has been of any use as a professional man, of any use to students or to his professional brethren, to this society belongs all the credit. He has helped to form other societies; this one formed him, and has a warmer place and a wider place in his heart than all the others.

Having become convinced both by experience and observation that the general practice of medicine gave a range too wide for thorough mental drill, and that, consequently, humanity would be better served by greater divisions of thought, or, in other words, by the practice of specialties, the convictions were reduced to practice, and Dental Surgery selected as the field of effort. New thoughts, new habits, new associations were inevitable. A feeling of bewilderment became oppressive. In such state of embarra-sment this society found me. I was a stranger; and it took me in. A sentence already quoted from the minutes may sound very tame to you all; but it records the turning point in the writer's whole professional life. "Drs. Watt and Hamill were present by invitation." That is all. All? Let us see.

An exchange of ideas with some of the members present at that meeting, and this mostly private social conversation gave them the opinion that I had paid some attention to the science of chemistry. The thought was still further developed at the next annual meeting, and the result was recognition as a teacher of chemistry in the Ohio College of Dental Surghry, an appointment for usefulness not often presented to one so young, or rather so new in any profession. How well this opportunity was improved is left to the judgment of others; but it is highly gratifying to the writer to reflect that ever since, with the exception of some two sessions, this department has been taught by the writer, or his chosen students. This, in a college which was the first to recognize chemistry as part of the curriculum of dental study, is no small honor, nor was it any less honor to succeed the Christian philospher, Dr. Slack, in this position.

Recognition of talent, however limited, development of character, and introduction to spheres of usefulness, seem to have been the special aims of this society. And this has been felt for good in many ways. In the deliberations of this society our mutual friend, Prof. J. Taft, first manifested the aptness to teach, which, by a few years development, became so fully recognized that he has been in demand in many fields of science, and is now the patriarch dental teacher, having merited this title by more years of continued service than any man living. But for the stimulating influence of this society in the early years of his professional career, he might have remained as quietly content with the broad, shaded streets of Xenia as the writer is at present dates.

Here, too, the mental energy of our friend Richardson acquired the momentum which gave us an invaluable text book. And time would fail to tell the many and valuable contributions to our periodical literature emanating from this society. Indeed many papers of special merit read in other societies were developed from thought and researches originating in this. The most carefully prepared paper ever furnished by the present writer was written at the special request of this society, though read in the American Dental Convention. I have no doubt other members present could bear similar testimony in reference to some of their own productions. The cases referred to are but specimens of development of professional character incident to proper associative effort. All honor, then, to the live, great, good men whose energy and forethought gave us such a blessing as this society.

But professional associations are now so common, it is not improbable that our younger brothers regard this as merely one of them, instead of reverencing her as the model mother of them all. Did time permit, it would be a pleasant duty to write out a list of her leading benefactions to the profession and to humanity. Naming a few, at random, is all that can be attempted now; and the writer is fully aware that many others, equally, if not more valuable, will rapidly pass through the minds of the older members. The establishment of the DENTAL REGISTER, and its support till able to walk alone, (as it has walked continuously to its thirtieth volume) is beyond all estimate in value. Continuous gum work begun and perfected within her pale; cylinder and block fillings; the swaged socket rim in plate work; the warm air syringe; greatly improved extracting forceps, (in co-operation with Mr. Sherwood;) the use of definite retaining points in filling; adhesive foil, (by Dr. Leslie); a change from the empirical to a rational use of local medicines on the dentine and pulp, starting directly from the discussions here in 1856; an essay here for popular reading, and the text books already mentioned in part. These are but parts of her ways; and a majority of these blessings were given us before the existence of most of the societies now actively co-operating with her. Truly may we say "Many daughters have done virtuously, but she excelleth them all."

Now in all that I have said, I have not endeavored to eulogize, nor have I endeavored to be egotistic. The thought all the time has been the great advantage of associative effort; and the developing influence of this association over myself and others has been used only to illustrate this thought. And if, by reading this, I have been able to persuade any of my younger brethren present that they can not run alone, but that they must have the co-operative sympathy and help of their professional brethren, my labor has its full reward. You can do no better than join this society. But if you do so, be a member in full communion. I enjoin this by word as I have done by example. I have been a member of the good old Mississippi Valley Association a quarter of a century, and in that time have failed to attend but two of its meetings, being prevented in both cases by severe illness.

A word to the young members and I close. Be prompt and faithful in attendance, free and easy in the discharge of membership duties, seek no position, and be sure that you are not slighted if not thrust into prominence. Though reaching the point of highest honor only at the close of a quarter of a century, I have been all the time petted, flattered, spoiled by this society; and for all her kindness I thank her.

